# Marine algal flora of Pico Island, Azores

**DOI:** 10.3897/BDJ.8.e57461

**Published:** 2020-10-01

**Authors:** Ana I. Azevedo Neto, Afonso C. L. Prestes, Nuno V. Álvaro, Roberto Resendes, Raul M. A. Neto, Ian Tittley, Ignacio Moreu

**Affiliations:** 1 cE3c - Centre for Ecology, Evolution and Environmental Changes/Azorean Biodiversity Group & Faculdade de Ciências e Tecnologia, Departamento de Biologia, Universidade dos Açores, 9500-321 Ponta Delgada, São Miguel, Açores, Portugal cE3c - Centre for Ecology, Evolution and Environmental Changes/Azorean Biodiversity Group & Faculdade de Ciências e Tecnologia, Departamento de Biologia, Universidade dos Açores 9500-321 Ponta Delgada, São Miguel, Açores Portugal; 2 Universidade dos Açores, Faculdade de Ciências Agrárias, CCMMG (Centro do Clima Meteorologia e Mudanças Globais), IITA-A (Instituto de Investigação e Tecnologias Agrárias e do Ambiente), Angra do Heroísmo, Terceira, Portugal Universidade dos Açores, Faculdade de Ciências Agrárias, CCMMG (Centro do Clima Meteorologia e Mudanças Globais), IITA-A (Instituto de Investigação e Tecnologias Agrárias e do Ambiente) Angra do Heroísmo, Terceira Portugal; 3 Universidade dos Açores, Faculdade de Ciências e Tecnologia, Departamento de Biologia, 9500-321 Ponta Delgada, São Miguel, Açores, Portugal Universidade dos Açores, Faculdade de Ciências e Tecnologia, Departamento de Biologia 9500-321 Ponta Delgada, São Miguel, Açores Portugal; 4 N/A, Odivelas, Portugal N/A Odivelas Portugal; 5 Natural History Museum, Cromwell Road, London, United Kingdom Natural History Museum Cromwell Road, London United Kingdom

**Keywords:** Macroalgae, Azores, Pico Island, new records, endemism, native, introduced, uncertain, occurrence data.

## Abstract

**Background:**

The seaweed flora of Pico Island (central group of the Azores archipelago) has attracted interest of researchers on past occasions. Despite this, the macroalgal flora of the island cannot be considered well-known as published information reflects only occasional collections. To overcome this, a thorough investigation encompassing collections and presence data recording was undertaken. Research under the Campaigns “AÇORES/89”, “PICO/91”, “PICOBEL/2007” and “LAUMACAT/2011” covered a relatively large area (approximately 39 km^2^) around the island, encompassing the littoral and sublittoral levels down to about 40 m around the Island.

This paper improves the knowledge of the Azorean macroalgal flora at local and regional scales by listing taxonomic records and providing information on the ecology and occurrence of each species present on the Island’s littoral.

**New information:**

A total of 4043 specimens (including taxa identified only to genus level) belonging to 303 taxa of macroalgae are registered, comprising 197 Rhodophyta, 53 Chlorophyta and 53 Ochrophyta (Phaeophyceae). From these, 225 were identified to species level (142 Rhodophyta, 41 Chlorophyta and 42 Ochrophyta), encompassing 110 new records for the island (69 Rhodophyta, 20 Chlorophyta and 21 Ochrophyta), three Macaronesian endemisms (*Botryocladia macaronesica* Afonso-Carillo, Sobrino, Tittley & Neto; *Laurencia viridis* Gil-Rodríguez & Haroun; *Codium elisabethiae* O. C. Schmidt), 14 introduced and 25 species with an uncertain status.

## Introduction

The Azorean algal flora, considered cosmopolitan with species shared with Macaronesia, North Africa, the Mediterranean Sea, Atlantic Europe and America (Tittley 2003, [Bibr B5880409][Bibr B5838454]), has been considered relatively rich when compared to that of other remote oceanic islands (Neto et al. 2005, Tittley and Neto 2005, Wallenstein et al. 2009). Even so, it is worth mentioning that the published information (approximately 400 species, Freitas et al. 2019) reflects data from only a few of the nine islands, since not all of them have been thoroughly investigated. To overcome this and improve the understanding of the archipelago’s seaweed flora, an effort has been made by local investigators over the past three decades and research on the marine macroalgae flora has been conducted on several of the less-studied Azorean islands. This paper comprises both physical and occurrence data and compiles the gathered information from macroalgae surveys developed in Pico Island mainly by the Island Aquatic Research Group of the University of the Azores (https://ce3c.ciencias.ulisboa.pt/sub-team/island-aquatic-ecology). It aims to constitute a practical resource for biological studies, such as systematics, diversity and conservation, biological monitoring, climate change and ecology and also for academics, students, government, private organisations and the general public.

## General description

### Purpose

By listing taxonomic records for Pico and presenting general information for each taxon occurrence on the Island’s littoral, this paper addresses several biodiversity shortfalls (see Cardoso et al. 2011, Hortal et al. 2015), namely the need to catalogue the Azorean macroalgae (Linnean shortfall) and improve the current information on their local and regional geographic distribution (Wallacean shortfall), as well as on species abundances and dynamics in space (Prestonian shortfall).

## Project description

### Title

Marine algal (seaweed) flora of Pico Island, Azores

### Personnel

Collections were conducted and occurrence data recorded during several years (1989-2018) under the coordination of Ana I. Neto. Main collectors were Abel Sentiés, Ana Costa, Ana I Neto, André Amaral, António Brigos, Catarina Santos, Daniel Torrão, David Villegas, Edgar Rosas-Alquicira, Edward Hehre, Emanuel Xavier, Eunice Nogueira, Francisco Wallenstein, Gustavo Martins, Heather Baldwin, Inês Neto, José M. N. Azevedo, Ian Tittley, Karla Leon Cisneros, Leila Bagaço, Maria Machín-Sánchez, Marlene Terra, Mutue Toyota Fujii, Nuno Álvaro, Patrícia Madeira, Raul Coma, Raul Neto, Richard Fralick, Rita Patarra, Ruben Afonso, Ruben Couto, Sílvia Escarduça and Valeria Cassano.

Several taxonomists contributed for the species identification: Abel Sentiés, Ana I. Neto, Edgar Rosas-Alquicira, Edward Hehre, Francisco Wallenstein, Heather Baldwin, Karla Leon Cisneros, Ian Tittley, Maria Machín-Sánchez, Marlene Terra, Mutue Toyota Fujii, Richard Fralick, Ruben Couto and Valeria Cassano.

Voucher specimen management was mainly undertaken by Afonso Prestes, Ana I. Neto, Eunice Nogueira, Natália Cabral and Roberto Resendes.

### Study area description

**Figure 1.**
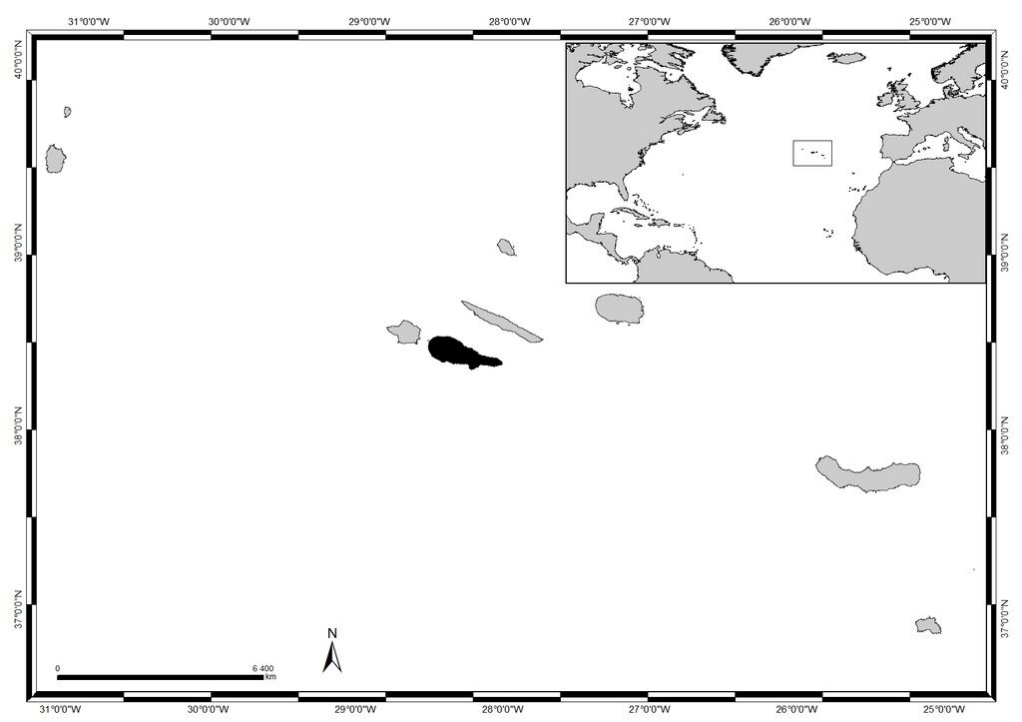
The Azores, its location in the Atlantic and Pico Island highlighted in black (by Nuno V. Álvaro).

Located in the North Atlantic, roughly at 38°43′49″N, 27°19′10″W, the Azores comprise nine islands and several islets spread over 500 km, in a WNW direction (Fig. 1). The climate is temperate oceanic, with regular and abundant rainfall, high levels of relative humidity and persistent winds, mainly during the winter and autumn seasons (Morton et al. 1998). The islands lack a continental shelf, thus presenting a restricted coastal extension and deep waters occur within a few kilometres offshore. Shore geomorphology varies considerably with high cliffs in some places and rocky cobble/boulder beaches elsewhere (Borges 2004), the tidal range is small (< 2 m, see Hidrográfico 1981) and coasts are subjected to swell and surge most of the year.

Pico (in black in Fig. 1), of approximately 447 km^2^ and dominated by its 2351 m tall mountain, is the second largest and the youngest island of the Azores archipelago, composed of basaltic volcanic deposits less than 300,000 years old (Cruz and Oliveira 2001). The Island's coastline is approximately 126 km long, generally devoid of high cliffs and consists mainly of irregular extensions of bedrock, presenting a variety of stack, arch and gully formations due to its recent volcanic origin. Intertidal platforms, occasionally dissected by channels and gullies, are mostly easily accessible by land and exhibit considerable variation in width (Wallenstein et al. 2009). Important features and habitats at this shore level are rock pools. These differ in shape and size and are a stressful environment due to the changes in salinity caused by either evaporation or dilution during low tides. They often recreate a shallow subtidal habitat and contain a rich diversity of marine life. A few shores consist of irregularly rounded boulders or cobles between which coarse sand or gravel may be retained. Sandy shores are rare (Neto pers. observ.).

**Figure 2.**
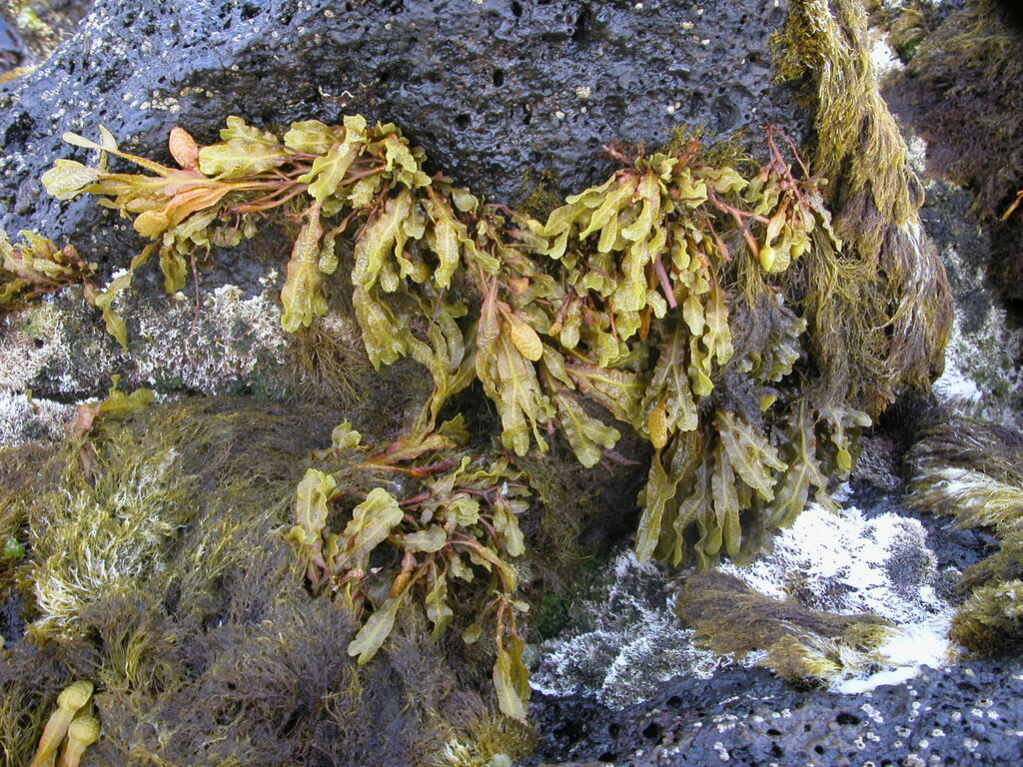
Littorinids, chthamalid barnacles and the algae *Fucus spiralis* and *Gelidium microdon* at the high intertidal (by the Island Aquatic Ecology Subgroup of cE3c-ABG).

**Figure 3.**
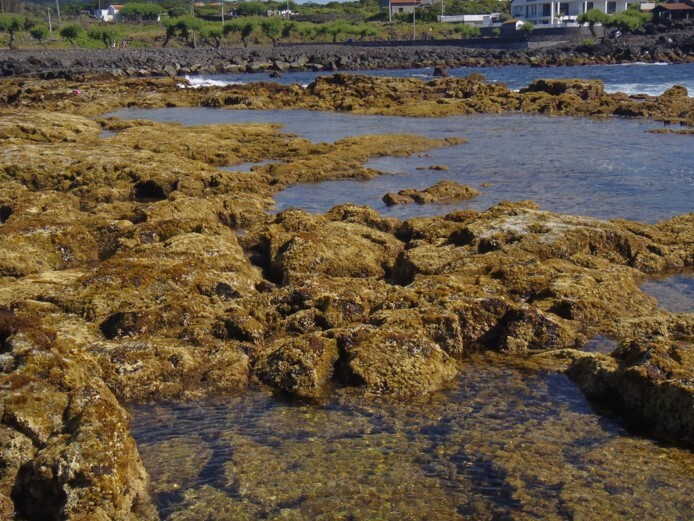
Mid-shore intertidal levels covered by algal turfs (by the Island Aquatic Ecology Subgroup of cE3c-ABG).

**Figure 4.**
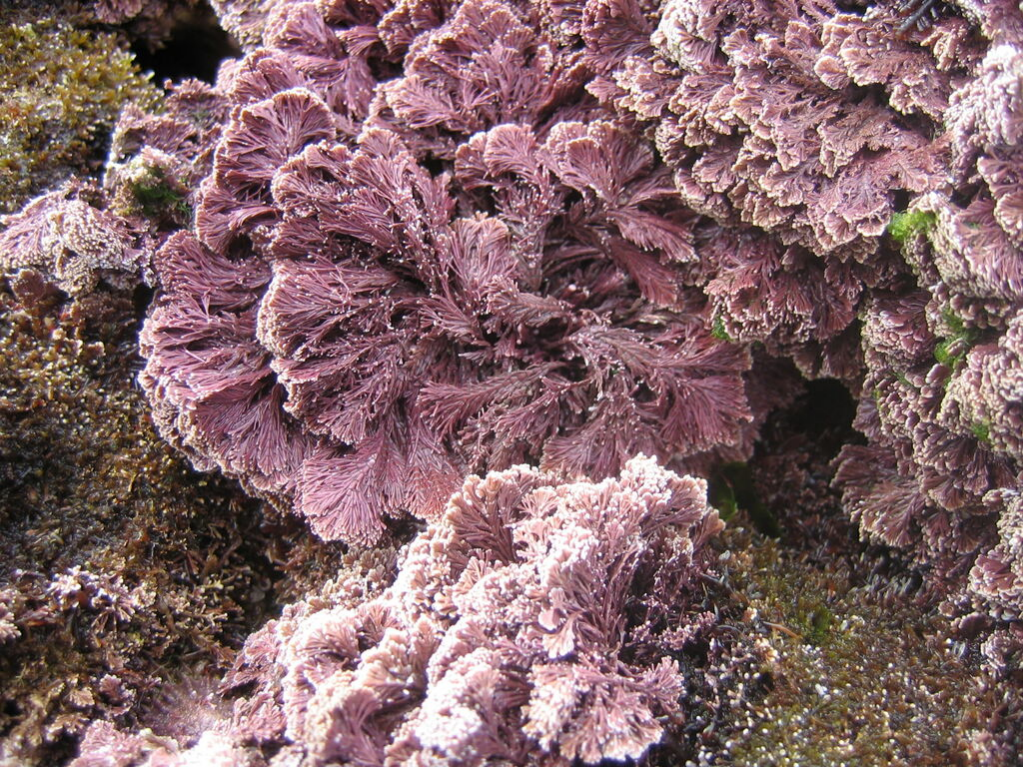
*Ellisolandia elongata* at lower intertidal levels (by the Island Aquatic Ecology Subgroup of cE3c-ABG).

**Figure 5.**
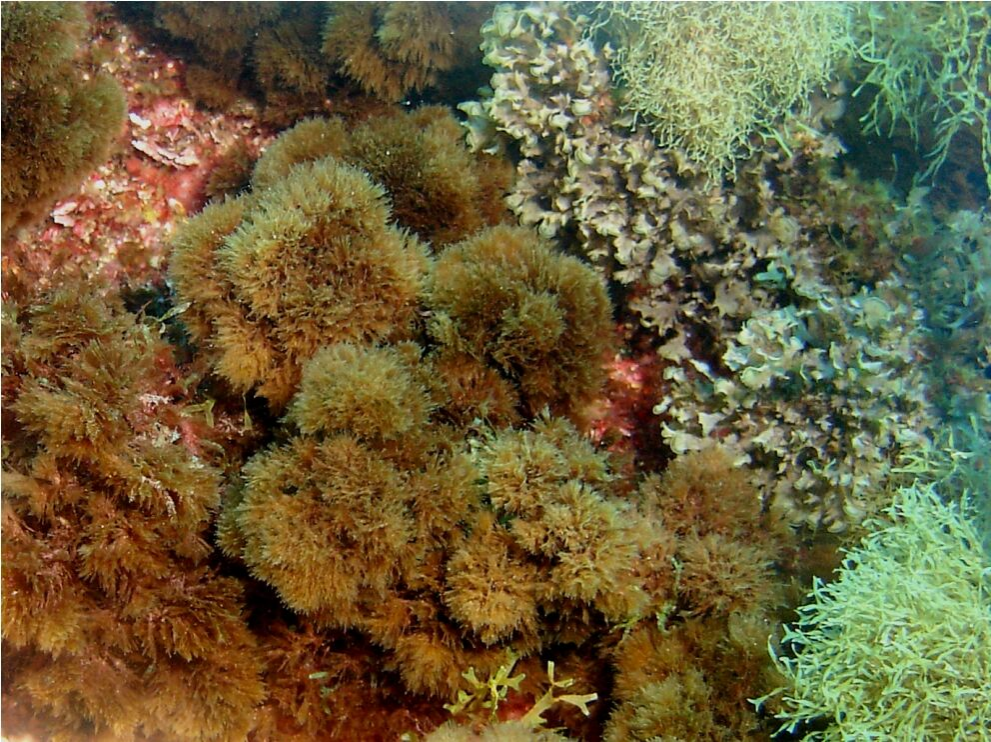
Frondose macrophytes (*Halopteris* spp., *Zonaria tournefortii* and *Dictyota* spp.) at the subtidal level (by the Island Aquatic Ecology Subgroup of cE3c-ABG).

The rocky-shore communities of Pico, like all over in the Azores, are dominated by macroalgae at both intertidal and shallow subtidal levels (Neto et al. 2005). At intertidal levels, a distinct zonation pattern is evident with a higher zone dominated by invertebrates (littorinids and chthamalid barnacles, in which patches or fringes of the algae *Fucus spiralis* Linnaeus and *Gelidium microdon* Kützing can occur (Fig. 2). This is followed by a mid-shore zone covered by algal turfs (growth forms of either diminutive algae or diminutive forms of larger species that create a dense, compact mat 20-30 mm thick, Fig. 3). Depending on the shore, turf can be monospecific (of either *Caulacanthus ustulatus* (Mertens ex Turner) Kützing, *Centroceras clavulatum* (C. Agardh) Montagne or *Gymnogongrus*) or multispecific and composed of soft algae (e.g. *Centroceras clavulatum*, *Chondracanthus* and *Laurencia*) usually growing as epiphytes over articulate calcareous forms (e.g. *Ellisolandia* and *Jania*). The lower zone is mainly dominated by calcareous crusts (first strata), covered by corticated macrophytes (e.g. *Ellisolandia elongata* (Fig. 4), *Pterocladiella capillacea*, *Treptacantha abies-marina* (S.G.Gmelin) Kützing). Mainly during spring and summer, considerable amounts of the introduced *Asparagopsis armata* Harvey can be seen at this level. Subtidally, algal communities are mainly characterised associations of two or three frondose macrophytes, for example, *Dictyota*, *Halopteris* and *Zonaria tournefortii* (J.V.Lamouroux) Montagne (Fig. 5).

### Design description

The algae referred to in this paper were collected during field studies at littoral and sublittoral levels down to approximately 40 m around Pico Island. Each sampling location was visited several times and, on each occasion, a careful survey was made covering much of the area. Presence/absence data were recorded for all known species and whenever an unknown or potential new species was found, it was collected, assigned an individual registration number and vouchers were deposited at the AZB Herbarium Ruy Telles Palhinha, based at the Faculty of Sciences and Technology of the University of the Azores.

### Funding

This study was mainly financed by the following projects/scientific expeditions:

- Campaign AÇORES/89, under the Expedition Azores/89, Departamento de Oceanografia e Pescas da Universidade dos Açores, July 1989;
- Expedition PICO/91, Ilha do Pico, Açores, Departamento de Biologia da Universidade dos Açores Ilha do Pico, Açores, June 1991;
- Campaign PICOBEL/2007, under the project “PICOBEL: Coastal benthic communities of Pico Island: characterization and monitoring”. 2007- 2008. The Azores Regional Government;
- Campaign LAUMACAT/2011 under the project “LAUMACAT: Diversity and phylogenetic relationships on the benthic marine algae with pharmacological potencial: the Laurencia complex (Rhodophyta) in Macaronesian archipelagos, tropical and subtropical Atlantic”. Phase II. 2011 - 2014. General Direction for Research and Management: Research Projects. Spanish Government;
- Project “ACORES-01-0145-FEDER-000072 - AZORES BIOPORTAL – PORBIOTA. Operational Programme Azores 2020 (85% ERDF and 15% regional funds);
- Portuguese National Funds, through FCT – Fundação para a Ciência e a Tecnologia, within the projects UID/BIA/00329/2013, 2015 - 2018 and UID/BIA/00329/2019 and UID/BIA/00329/2020-2023;
- Portuguese Regional Funds, through DRCT – Direção Regional da Ciência e Tecnologia, within several projects, since 2013;
- CIRN/DB/UAc (Research Centre for Natural Resources, Universidade dos Açores, Departamento de Biologia);
- CIIMAR (Interdisciplinary Centre of Marine and Environmental Research, Porto, Portugal).

## Sampling methods

### Study extent

**Figure 6.**
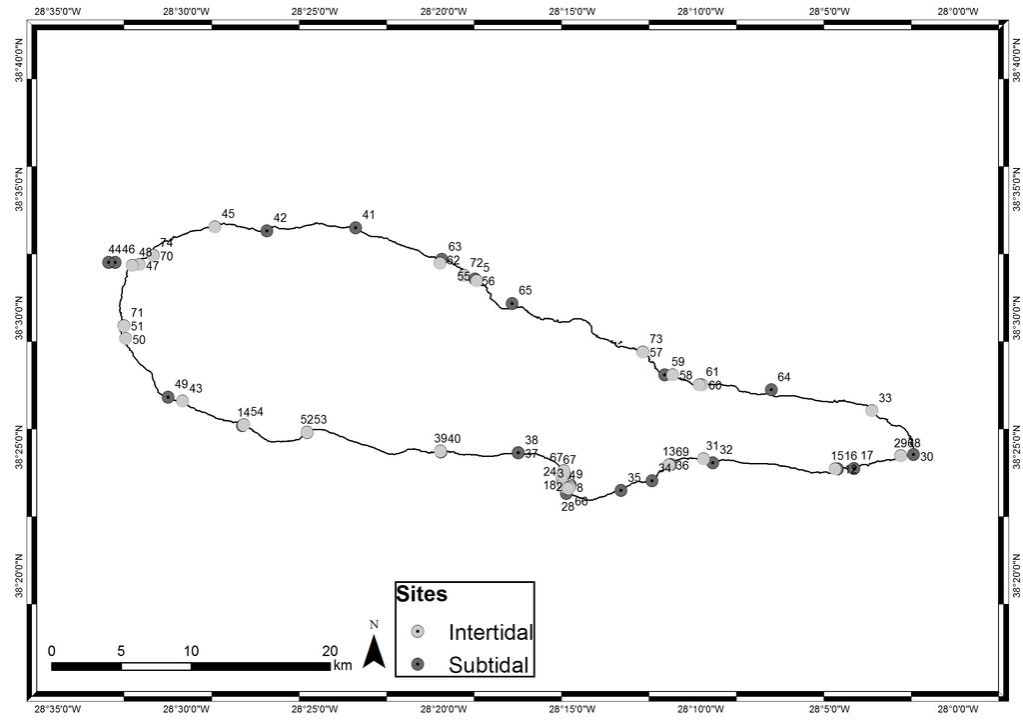
Sampling locations around Pico Island (by Nuno V. Álvaro).

This study covers a relatively large area, approximately 39 km^2^, encompassing littoral and sublittoral levels down to approximately 40 m around the island (Table 1, Fig. 6).

### Sampling description

**Figure 7.**
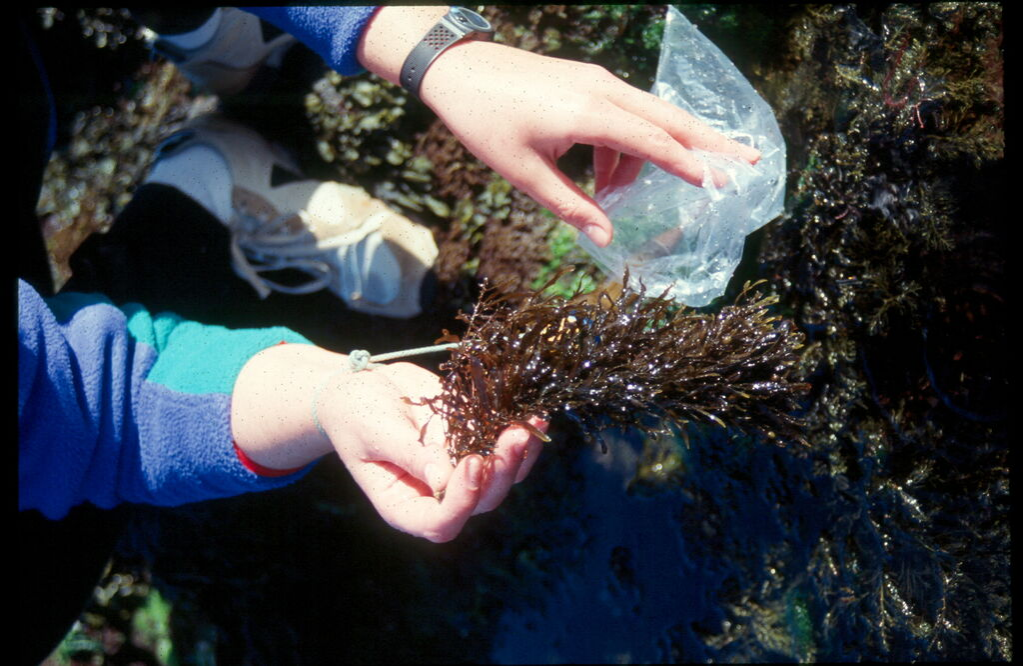
Collecting macroalgae at the rocky intertidal (by the Island Aquatic Ecology Subgroup of cE3c-ABG).

**Figure 8.**
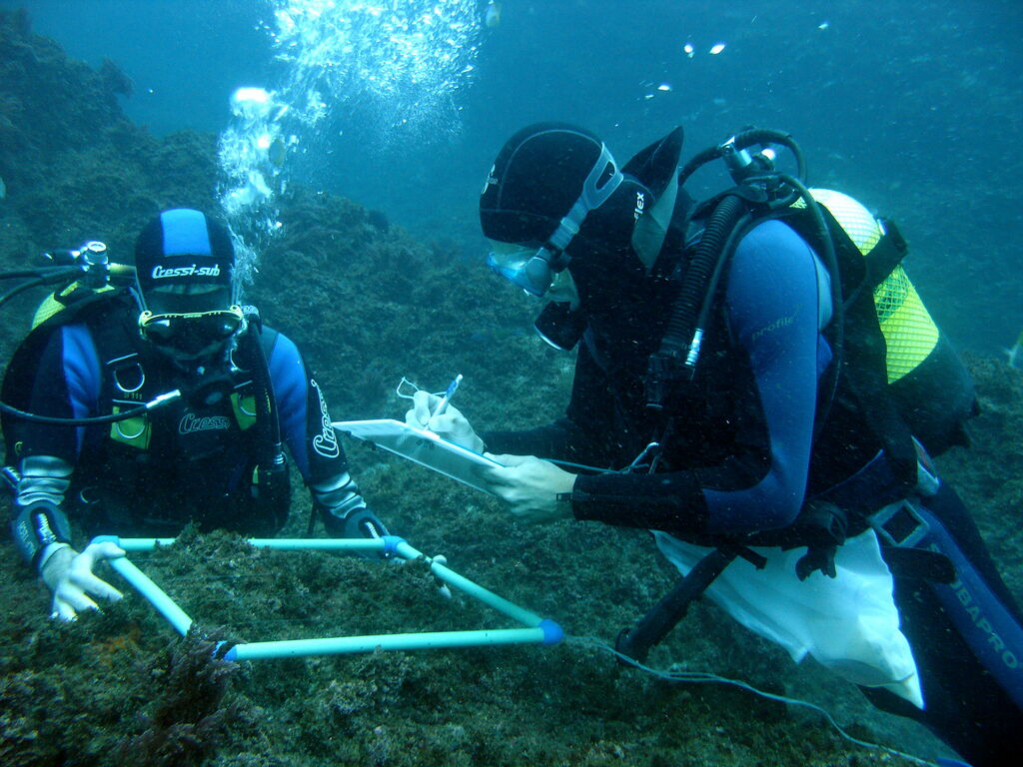
Subtidal species recording data (by the Island Aquatic Ecology Subgroup of cE3c-ABG).

Intertidal collections were made at low tide by walking over the shores. Subtidal collections were made by scuba diving around the area. Sampling encompassed both physical collections and species presence recordings. For the former, in each sampling location, collections were made manually by scraping one or two specimens of species found into previously labelled bags (Fig. 7). Species recording data were gathered by registering all species present in the sampled locations visited (Fig. 8). Complementary data, for example, shore level (high, mid, low), orientation and type of substrate (bedrock, boulders, cobbles, mixed), habitat (tide pool, open rock, gully, crevice, cave) were also recorded.

### Quality control

Each sampled taxon was investigated by trained taxonomists with the help of keys and floras. This involved morphological and anatomical examination by eye or under dissecting and compound microscopes of an entire specimen or slide preparation. In difficult cases, specimens were sent to experts for identification.

### Step description

In the laboratory, the specimens were sorted and studied following standard procedures used in macroalgae identification.

Species identification was based on morphological and anatomical characters and reproductive structures. For small and simple thalli, this required the observation of the entire thallus by eye and/or under dissecting or compound microscopes. For larger and more complex algae, the investigation of the thallus anatomy required histological work to obtain longitudinal and transverse sections needed for the observation of cells, reproductive structures and other diagnosing characters.

The Azorean algal flora has components from several geographical regions which makes for difficulties in identification. Floras and identification keys to macroalgae in the Atlantic and Western Mediterranean were used in species identification (e.g. Schmidt 1931, Taylor 1967, Taylor 1978, Levring 1974, Dixon and Irvine 1977, Lawson and John 1982, Irvine 1983, Irvine and Chamberlain 1994, Gayral and Cosson 1986, Fletcher 1987, Afonso-Carrillo and Sansón 1989, Burrows 1991, Boudouresque et al. 1992, Cabioc'h et al. 1992, Maggs and Hommersand 1993, Irvine and Chamberlain 1994, Irvine and Chamberlain 1994, Brodie et al. 2007, Lloréns et al. 2012, Rodríguez-Prieto et al. 2013).

For more critical and taxonomically-difficult taxa, specimens were taken to the Natural History Museum (London) for comparison with collections there or sent to appropriate specialists.

A reference collection was made for all collected specimens by assigning them a herbarium code number and depositing them at the AZB Herbarium Ruy Telles Palhinha, University of Azores. Depending on the species and on planned further research, different types of collections were made, namely (i) liquid collections using 5% buffered formaldehyde seawater and then replacing it by the fixing agent Kew (Bridsen and Forman 1999); (ii) dried collections, either by pressing the algae (most species) as described by Gayral and Cosson 1986) or by letting them air dry (calcareous species); (iii) silica collections for molecular studies.

Nomenclatural and taxonomic status used here follow *Algaebase* (Guiry and Guiry 2020). The database was organised on FileMaker Pro.

## Geographic coverage

### Description

Pico Island, Azores, Macaronesia, Portugal (approximately 38°34'02″N, 28°33′17″W).

### Coordinates

38.376 and 38.567 Latitude; -28.555 and -28.021 Longitude.

## Taxonomic coverage

### Description

All macroalgae were identified to genus or species. In total, 303 taxa were identified belonging to 30 orders and 67 families, distributed by the phyla Rhodophyta (15 orders and 39 families), Chlorophyta (3 orders and 10 families) and Ochrophyta (12 orders and 18 families).

### Taxa included

**Table:** 

Rank	Scientific Name	Common Name
phylum	Rhodophyta	Red algae
phylum	Chlorophyta	Green algae
phylum	Ochrophyta	Brown algae

## Temporal coverage

**Data range:** 1989-5-01 – 2018-9-30.

### Notes

The sampling was performed on several occasions in the period between 1989 and 2018.

## Collection data

### Collection name

AZB | Marine macroalgae collection of Pico Island (Azores) – Expedition AZORES/89; AZB | Marine macroalgae collection of Pico Island (Azores) – Expedition PICO/91; AZB | Marine macroalgae collection of Pico Island (Azores) – Project PICOBEL; AZB | Marine macroalgae collection of Pico Island (Azores) – Project LAUMACAT; AZB | Marine macroalgae collection of Pico Island (Azores) – Occasional sampling; Marine macroalgae occurrence in Pico Island (Azores) – Expedition AZORES/89; Marine macroalgae occurrence in Pico Island (Azores) – Project PICOBEL; Marine macroalgae occurrence in Pico Island (Azores) – Project LAUMACAT.

### Collection identifier

4ea1e09c-13c8-4b8e-a28a-72c55bde8f66; 0f2368fa-0a53-43c5-9f19-b126260e4e83; 6163248c-236b-4778-99cf-39dbf28a9784; b4ed4e44-3e8f-42d4-a44b-d78585a8f8f0; acc4fc70-0cb6-496e-982c-9207d09b856a; 468e613d-1ce9-4a32-a5f9-c5a8b58545c1; a8405f3e-fdc6-452d-9dc9-ca1fd3abdf2c; 84ff06f5-8c7c-4c3c-9296-38ad24b347bd.

### Parent collection identifier

AZB Herbarium Ruy Telles Palhinha, Faculty of Sciences and Technology of the University of the Azores.

### Specimen preservation method

Air dry, Dried and pressed; Liquid (Formalin; fixing agent Kew), Silica.

### Curatorial unit

AZB Herbarium Ruy Telles Palhinha, Faculty of Sciences and Technology of the University of the Azores.

## Usage rights

### Use license

Open Data Commons Attribution License

### IP rights notes

Creative Commons Attribution (CC-BY) 4.0 License

## Data resources

### Data package title

Marine algal flora of Pico Island, Azores

### Resource link

http://ipt.gbif.pt/ipt/resource?r=pico_seaweed_flora&v=1.11

### Alternative identifiers

https://www.gbif.org/dataset/6af010f0-8238-4745-8309-21c3f82bd488

### Number of data sets

1

### Data set 1.

#### Data set name

Marine algal (seaweed) flora of Pico Island, Azores

#### Data format

Darwin Core Archive

#### Number of columns

51

#### Download URL

http://ipt.gbif.pt/ipt/resource?r=pico_seaweed_flora

#### Data format version

v1.11

#### Description

This data paper presents physical and occurrence data from macroalgal surveys undertaken on Pico Island between 1989 and 2018. The dataset submitted to GBIF is structured as a sample event dataset, with two tables: event (as core) and occurrences (Neto et al. 2020). The data in this sampling event resource have been published as a Darwin Core Archive (DwCA), which is a standardised format for sharing biodiversity data as a set of one or more data tables. The core data table contains 74 records (eventID). The extension data table has 4043 occurrences. An extension record supplies extra information about a core record. The number of records in each extension data table is given in the IPT link. This IPT archives the data and thus serves as the data repository. The data and resource metadata are available for downloading in the downloads section.

**Data set 1.** 

Column label	Column description
Table of Sampling Events	Table with sampling events data (beginning of table)
eventID	Identifier of the event, unique for the dataset
country	Country of the sampling site
countryCode	Code of the country where the event occurred
stateProvince	Name of the region
island	Name of the island
municipality	Name of the municipality
locality	Name of the locality
locationID	Identifier of the location
decimalLatitude	The geographic latitude of the sampling site
decimalLongitude	The geographic longitude of the sampling site
geodeticDatum	The spatial reference system upon which the geographic coordinates are based
coordinateUncertaintyInMetres	The horizontal distance (in metres) from the given decimalLatitude and decimalLongitude describing the smallest circle containing the whole of the Location
eventDate	Time interval when the event occurred
year	The year of the event
samplingProtocol	Sampling method used during an event
locationRemarks	Zonation level
minimumDepthInMetres	The minimum depth in metres where the specimen was found
maximumDepthInMetres	The maximum depth in metres where the specimen was found
eventRemarks	Notes about the event
Table of Species Occurrence	Table with species occurrence data (beginning of new table)
occurrenceID	Identifier of the record, coded as a global unique identifier
institutionID	The identifier for the institution having custody of the object or information referred to in the record
institutionCode	The acronym of the institution having custody of the object or information referred to in the record
collectionID	An identifier of the collection to which the record belongs
collectionCode	The name of the collection from which the record was derived
datasetName	The name identifying the dataset from which the record was derived
eventID	Identifier of the event, unique for the dataset
kingdom	Kingdom name
phylum	Phylum name
class	Class name
order	Order name
family	Family name
genus	Genus name
specificEpithet	The name of the first or species epithet of the scientificName
infraspecificEpithet	The name of the lowest or terminal infraspecific epithet of the scientificName, excluding any rank designation
acceptedNameUsage	The specimen accepted name, with authorship
previousIdentifications	Previous name of the specimen, with authorship
scientificName	The name without authorship applied on the first identification of the specimen
basisOfRecord	The specific nature of the data record
habitat	Description of the habitat where the specimen was found
organismQuantityType	The type of quantification system used to quantity the organisms
organismQuantity	Percentage of the organism coverage
recordedBy	Person(s) responsible for sampling
catalogNumber	Identifying code for a unique sample lot in a biological collection
identifiedBy	Person(s) responsible for taxa identification
type	The nature of the resource
preparations	The preservation method used for the specimen
establishmentMeans	The establishment status of the organism in the study region
occurrenceRemarks	New record status assignment
licence	Reference to the licence under which the record is published

## Additional information

**Figure 9.**
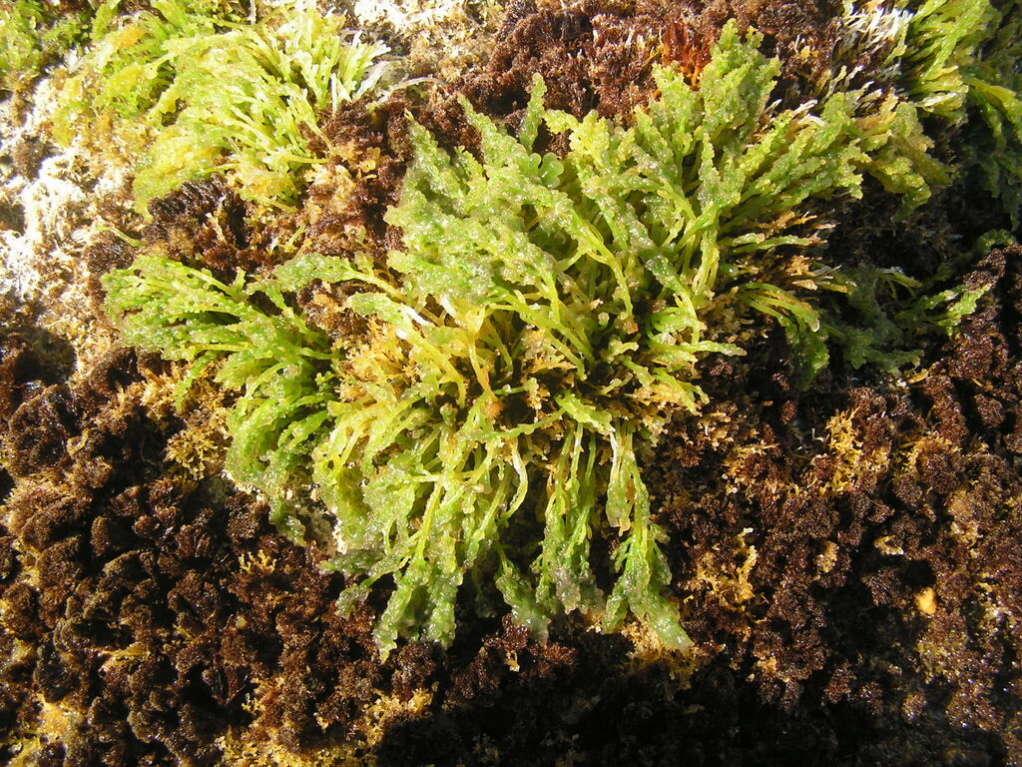
*Laurencia pyramidalis*, a new record for Pico Island (by the Island Aquatic Ecology Subgroup of cE3c-ABG).

This paper accommodates the 4043 specimens of macroalgae recorded from Pico Island in 303 taxa comprising 225 confirmed species and 78 taxa identified only to genus level. The confirmed species (Tables 2, 3) include 142 Rhodophyta, 41 Chlorophyta and 42 Ochrophyta (Phaeophyceae). From these, 110 species are newly-recorded to the island (69 Rhodophyta, 20 Chlorophyta and 21 Ochrophyta), as, for example, *Laurencia pyramidalis* Bory ex Kützing (Fig. 9). Most species are native, including the three Macaronesian endemisms *Botryocladia macaronesica* Afonso-Carillo, Sobrino, Tittley & Neto, *Laurencia viridis* Gil-Rodríguez & Haroun and *Codium elisabethiae* O. C. Schmidt. Fourteen species represent introductions to the algal flora and 25 have an uncertain status.

Many species were only sporadically recorded on Pico, but 11 were commonly found around the Island and occurred quite abundantly in some locations: the Rhodophyta *Asparagopsis armata* Harvey, *Ellisolandia elongata* (J. Ellis & Solander) K. R. Hind & G. W. Saunders, *Hypnea musciformis* (Wulfen) J. V. Lamouroux and *Pterocladiella capillacea* (S. G. Gmelin) Santelices & Hommersand; the Chlorophyta *Anadyomene saldanhae* A. B. Joly & E. C. Oliveira, *Codium adhaerens* C. Agradh and *Ulva rigida* C. Agardh; the Ochrophyta *Colpomenia sinuosa* (Mertens ex Roth) Derbès & Solier in Castagne, *Halopteris scoparia* (Linnaeus) Sauvageau, *Padina pavonica* (Linnaeus) Thivy and *Zonaria tournefortii* (J. V. Lamouroux) Montagne.

A mismatch regarding the GBIF backbone taxonomy of some of the macroalgae species names was identified as detailed in Suppl. material 1.

## Supplementary Material

0EC9DB15-B145-57EE-ADED-311D06B9F1EC 10.3897/BDJ.8.e57461.suppl1 Supplementary material 1 DP-PIX-id_14106_normalized-redz.csv Data type Macroalgae taxonomic mismatching Brief description GBIF does not have the more actualised nomenclature for some of the macroalgae species names. Therefore, the matching tools of its platform were applied to the species list, as required by Pensoft's data auditor, to identify the problematic taxonomic situations. The resulting file (DP-PIX-id_14106_normalized-redz.csv) is included here, since the names will not be immediately updated in the GBIF Taxonomic Backbone. A request was already sent to GBIF helpdesk to resolve this situation. File: oo_438159.csv https://binary.pensoft.net/file/438159 Ana I. Neto

## Figures and Tables

**Table 1.** Pico Island sampling sites information.

Location N0	Location ID	Municipality	Locality	Latitude / Longitude	Littoral zone
1	PIX_LP_LPlac	Lajes do Pico	Lajes do Pico|Lagoa de Cima	38.398018, -28.254506	Subtidal
2	PIX_LP_LPMbsp	Lajes do Pico	Lajes do Pico|Maré|Baía de S. Pedro	38.391826, -28.251878	Intertidal
3	PIX_LP_LPMbsp	Lajes do Pico	Lajes do Pico|Maré|Baía de S. Pedro	38.391602, -28.251087	Subtidal
4	PIX_LP_LPMpb	Lajes do Pico	Lajes do Pico|Maré|Ponta da Barra	38.389773, -28.25225	Intertidal
5	PIX_SR_CPpi	São Roque	Cais do Pico|Piscina	38.523888, -28.31165	Intertidal
6	PIX_LP_LPLa	Lajes do Pico	Lajes do Pico|Lagido	38.39961, -28.255085	Intertidal
7	PIX_LP_LPLa	Lajes do Pico	Lajes do Pico|Lagido	38.399708, -28.255538	Subtidal
8	PIX_LP_LPMbsp	Lajes do Pico	Lajes do Pico|Maré|Baía de S. Pedro	38.391602, -28.251087	Subtidal
9	PIX_LP_LPMpb	Lajes do Pico	Lajes do Pico|Maré|Ponta da Barra	38.390439, -28.251473	Intertidal
10	PIX_LP_LPMpp	Lajes do Pico	Lajes do Pico|Maré|Poça do Pano	38.390439, -28.251473	Intertidal
11	PIX_LP_LPMpp	Lajes do Pico	Lajes do Pico|Maré|Poça do Pano	38.390439, -28.251473	Subtidal
12	PIX_LP_CNpo	Lajes do Pico	Calheta do Nesquim|Porto	38.402234, -28.078158	Subtidal
13	PIX_LP_SCp	Lajes do Pico	Santa Cruz|Porto	38.405086, -28.185822	Subtidal
14	PIX_MA_SMp	Madalena	São Mateus|Porto	38.43023, -28.463406	Subtidal
15	PIX_LP_CNpo	Lajes do Pico	Calheta do Nesquim|Porto	38.40219, -28.079374	Intertidal
16	PIX_LP_CNpo	Lajes do Pico	Calheta do Nesquim|Porto	38.402234, -28.078158	Subtidal
17	PIX_LP_Fc	Lajes do Pico	Feteira|Calheta	38.402428, -28.067472	Subtidal
18	PIX_LP_LPc1	Lajes do Pico	Lajes do Pico|Controlo 1	38.395374, -28.256297	Intertidal
19	PIX_LP_LPc2	Lajes do Pico	Lajes do Pico|Controlo 2	38.392892, -28.255585	Intertidal
20	PIX_LP_LPc3	Lajes do Pico	Lajes do Pico|Controlo 3	38.390764, -28.255038	Intertidal
21	PIX_LP_LPc4	Lajes do Pico	Lajes do Pico|Controlo 4	38.392892, -28.255585	Intertidal
22	PIX_LP_LPc5	Lajes do Pico	Lajes do Pico|Controlo 5	38.392892, -28.255585	Intertidal
23	PIX_LP_LPc6	Lajes do Pico	Lajes do Pico|Controlo 6	38.392892, -28.255585	Intertidal
24	PIX_LP_LPfb	Lajes do Pico	Lajes do Pico|Fabrica baleia	38.398098, -28.255913	Intertidal
25	PIX_LP_LPLa	Lajes do Pico	Lajes do Pico|Lagido	38.39961, -28.255085	Intertidal
26	PIX_LP_LPMpb	Lajes do Pico	Lajes do Pico|Maré|Ponta da Barra	38.38958, -28.252883	Subtidal
27	PIX_LP_LPMpp	Lajes do Pico	Lajes do Pico|Maré|Poça do Pano	38.390439, -28.251473	Intertidal
28	PIX_LP_LPpc	Lajes do Pico	Lajes do Pico|Ponta do Castelete	38.386303, -28.253616	Subtidal
29	PIX_LP_Pm	Lajes do Pico	Piedade|Manhenha	38.410566, -28.036926	Intertidal
30	PIX_LP_Pm	Lajes do Pico	Piedade|Manhenha	38.41154, -28.028848	Subtidal
31	PIX_LP_PN	Lajes do Pico	Pontas Negras	38.408753, -28.164838	Intertidal
32	PIX_LP_PN	Lajes do Pico	Pontas Negras	38.406176, -28.158753	Subtidal
33	PIX_LP_Ppc	Lajes do Pico	Piedade|Porto do Calhau	38.439663, -28.055835	Intertidal
34	PIX_LP_SB	Lajes do Pico	Santa Bárbara	38.394346, -28.198255	Subtidal
35	PIX_LP_Sbpa	Lajes do Pico	Santa Bárbara|Ponta do Arrife	38.388247, -28.218223	Subtidal
36	PIX_LP_SCpi	Lajes do Pico	Santa Cruz|Piscinas	38.404764, -28.187053	Intertidal
37	PIX_LP_Sf	Lajes do Pico	Silveira|Fonte	38.412468, -28.284707	Subtidal
38	PIX_LP_Sf	Lajes do Pico	Silveira|Fonte	38.412468, -28.284707	Subtidal
39	PIX_LP_SJp	Lajes do Pico	São João|Porto	38.413619, -28.335026	Intertidal
40	PIX_LP_SJp	Lajes do Pico	São João|Porto	38.41324, -28.334801	Subtidal
41	PIX_MA_CA	Madalena	Cabrito	38.55817, -28.390065	Subtidal
42	PIX_MA_CAC	Madalena	Cachorro	38.556193, -28.44759	Subtidal
43	PIX_MA_GU	Madalena	Guindaste	38.445879, -28.502033	Intertidal
44	PIX_MA_MAc	Madalena	Madalena|Canal	38.535844, -28.549904	Subtidal
45	PIX_MA_MAcm	Madalena	Madalena|Cais do Mourato	38.558712, -28.481251	Intertidal
46	PIX_MA_MAi	Madalena	Madalena|Ilhéus	38.535854, -28.545742	Subtidal
47	PIX_MA_MAp	Madalena	Madalena|Porto	38.534214, -28.530273	Intertidal
48	PIX_MA_MApi	Madalena	Madalena|Piscina	38.533837, -28.534756	Intertidal
49	PIX_MA_mi	Madalena	Mirateca	38.448621, -28.511449	Subtidal
50	PIX_MA_MOpc	Madalena	Monte|Porto do Calhau	38.486345, -28.538979	Intertidal
51	PIX_MA_MOpo	Madalena	Monte|Pocinho	38.494812, -28.540192	Intertidal
52	PIX_MA_SCp	Madalena	São Caetano|Porto	38.425816, -28.421585	Intertidal
53	PIX_MA_SCp	Madalena	São Caetano|Porto	38.425656, -28.421382	Subtidal
54	PIX_MA_SMp	Madalena	São Mateus|Porto	38.430824, -28.462433	Intertidal
55	PIX_SR_CPpi	São Roque	Cais do Pico|Piscina	38.523888, -28.31165	Intertidal
56	PIX_SR_CPpi	São Roque	Cais do Pico|Piscina	38.52502, -28.312802	Subtidal
57	PIX_SR_PrN	São Roque	Prainha do Norte	38.477785, -28.204158	Intertidal
58	PIX_SR_PrNbc	São Roque	Prainha do Norte|Baia de Canas	38.462872, -28.184805	Intertidal
59	PIX_SR_PrNca	São Roque	Prainha do Norte|Canto da Areia	38.462994, -28.19003	Subtidal
60	PIX_SR_SAbc	São Roque	Santo Amaro|Baía do Canto	38.456384, -28.165936	Intertidal
61	PIX_SR_SAc	São Roque	Santo Amaro|Caisinho	38.456591, -28.16742	Intertidal
62	PIX_SR_SAnt	São Roque	Santo António	38.535259, -28.335402	Intertidal
63	PIX_SR_SAnt	São Roque	Santo António	38.537581, -28.334334	Subtidal
64	PIX_SR_SApm	São Roque	Santo Amaro|Ponta do Mistério	38.45332, -28.120977	Subtidal
65	PIX_SR_SMA	São Roque	São Miguel Arcanjo	38.509128, -28.288709	Subtidal
66	PIX_LP_LPMppb	Lajes do Pico	Lajes do Pico|Maré|Ponta da poça da baleia	38.389725, -28.252395	Intertidal
67	PIX_LP_LPp	Lajes do Pico	Lajes do Pico|Portinho	38.400994, -28.25526	Intertidal
68	PIX_LP_Pm	Lajes do Pico	Piedade|Manhenha	38.410566, -28.036926	Intertidal
69	PIX_LP_SCpi	Lajes do Pico	Santa Cruz|Piscinas	38.404764, -28.187053	Intertidal
70	PIX_MA_MAb	Madalena	Madalena|Barca	38.540254, -28.521195	Intertidal
71	PIX_MA_MOpo	Madalena	Monte|Pocinho	38.494812, -28.540192	Intertidal
72	PIX_SR_CPpov	São Roque	Cais do Pico|Porto Velho	38.527148, -28.319716	Intertidal
73	PIX_SR_PrN	São Roque	Prainha do Norte	38.477785, -28.204158	Intertidal
74	PIX_MA_MAb	Madalena	Madalena|Barca	38.540254, -28.521195	Intertidal

**Table 2.** Macroalgae species from Pico Island, with information on number of records, biogeographic origin and occurrence remarks (A: abundant; C: common; R: rare).

**Phylum**	**Species (Accepted Name)**	**Number of records**	**Establishment Means**	**Occurrence Remarks**	
Rhodophyta	*Acrosorium ciliolatum* (Harvey) Kylin	34	Native	New record	C
Rhodophyta	*Aglaothamnion cordatum* (Børgesen) Feldmann-Mazoyer	1	Introduced		R
Rhodophyta	*Aglaothamnion tenuissimum* (Bonnemaison) Feldmann-Mazoyer	2	Uncertain		R
Rhodophyta	*Ahnfeltiopsis devoniensis* (Greville) P.C.Silva & DeCew	5	Native	New record	R
Rhodophyta	*Amphiroa beauvoisii* J.V.Lamouroux	11	Native		C
Rhodophyta	*Amphiroa fragilissima* (Linnaeus) J.V.Lamouroux	2	Native	New record	R
Rhodophyta	*Amphiroa rigida* J.V.Lamouroux	4	Native	New record	R
Rhodophyta	*Anotrichium barbatum* (C.Agardh) Nägeli	1	Native	New record	R
Rhodophyta	*Anotrichium furcellatum* (J.Agardh) Baldock	1	Uncertain		R
Rhodophyta	*Antithamnion decipiens* (J.Agardh) Athanasiadis	4	Native	New record	R
Rhodophyta	*Antithamnion diminuatum* Wollaston	2	Introduced		R
Rhodophyta	*Antithamnionella boergesenii* (Cormaci & G.Furnari) Athanasiadis	2	Uncertain		R
Rhodophyta	*Aphanocladia stichidiosa* (Funk) Ardré	4	Native		R
Rhodophyta	*Asparagopsis armata* Harvey, phase *Falkenbergia rufolanosa* (Harvey) F.Schmitz	42	Introduced	New record	A
Rhodophyta	*Asparagopsis armata* Harvey	142	Introduced		C
Rhodophyta	*Asparagopsis taxiformis* (Delile) Trevisan	35	Native		C
Rhodophyta	*Asteromenia peltata* (W.R.Taylor) Huisman & A.J.K.Millar	16	Native	New record	C
Rhodophyta	*Bornetia secundiflora* (J.Agardh) Thuret	3	Native	New record	R
Rhodophyta	*Botryocladia botryoides* (Wulfen) Feldmann	3	Native	New record	R
Rhodophyta	*Botryocladia macaronesica* Afonso-Carillo, Sobrino, Tittley & Neto	1	Macaronesian endemism	New record	R
Rhodophyta	*Callithamnion corymbosum* (Smith) Lyngbye	5	Native	New record	R
Rhodophyta	*Callithamnion granulatum* (Ducluzeau) C.Agardh	1	Native	New record	R
Rhodophyta	*Callithamnion tetragonum* (Withering) S.F.Gray	4	Native		R
Rhodophyta	*Carradoriella denudata* (Dillwyn) A.M.Savoie & G.W.Saunders	20	Uncertain		C
Rhodophyta	*Carradoriella elongata* (Hudson) A.M.Savoie & G.W.Saunders	9	Native	New record	R
Rhodophyta	*Catenella caespitosa* (Withering) L.M.Irvine in Parke & P.S.Dixon	1	Native	New record	R
Rhodophyta	*Caulacanthus ustulatus* (Mertens ex Turner) Kützing	10	Uncertain		C
Rhodophyta	*Centroceras clavulatum* (C.Agardh) Montagne	57	Native		A
Rhodophyta	*Ceramium ciliatum* (J.Ellis) Ducluzeau	18	Native		C
Rhodophyta	*Ceramium cingulatum* Weber Bosse	1	Introduced		R
Rhodophyta	*Ceramium circinatum* (Kützing) J.Agardh	1	Native	New record	R
Rhodophyta	*Ceramium codii* (H.Richards) Mazoyer	2	Native		R
Rhodophyta	*Ceramium diaphanum* (Lightfoot) Roth	21	Native		C
Rhodophyta	*Ceramium echionotum* J.Agardh	3	Native		R
Rhodophyta	*Ceramium gaditanum* (Clemente) Cremades	4	Uncertain		R
Rhodophyta	*Ceramium virgatum* Roth	42	Native		C
Rhodophyta	*Champia parvula* (C.Agardh) Harvey	3	Native		R
Rhodophyta	*Chondracanthus acicularis* (Roth) Fredericq	58	Native		A
Rhodophyta	*Chondracanthus teedei* (Mertens ex Roth) Kützing	26	Native	New record	C
Rhodophyta	*Chondria capillaris* (Hudson) M.J.Wynne	15	Native	New record	C
Rhodophyta	*Chondria coerulescens* (J.Agardh) Sauvageau	1	Uncertain		R
Rhodophyta	*Chondria dasyphylla* (Woodward) C.Agardh	13	Uncertain		C
Rhodophyta	*Coelothrix irregularis* (Harvey) Børgesen	1	Native		R
Rhodophyta	*Corallina officinalis* Linnaeus	5	Native		R
Rhodophyta	*Cottoniella filamentosa* (M.Howe) Børgesen	5	Native	New record	R
Rhodophyta	*Crouania attenuata* (C.Agardh) J.Agardh	11	Native		C
Rhodophyta	*Cryptopleura ramosa* (Hudson) L.Newton	6	Native		R
Rhodophyta	*Dasya corymbifera* J.Agardh	7	Native		R
Rhodophyta	*Dasya rigidula* (Kützing) Ardissone	2	Native	New record	R
Rhodophyta	*Dermocorynus dichotomus* (J.Agardh) Gargiulo, M.Morabito & Manghisi	63	Native		A
Rhodophyta	*Diplothamnion jolyi* C.Hoek	3	Native	New record	R
Rhodophyta	*Dudresnaya verticillata* (Withering) Le Jolis	1	Native	New record	R
Rhodophyta	*Ellisolandia elongata* (J.Ellis & Solander) K.R.Hind & G.W.Saunders	190	Native		A
Rhodophyta	*Erythrocystis montagnei* (Derbès & Solier) P.C.Silva	11	Native	New record	C
Rhodophyta	*Gaillona hookeri* (Dillwyn) Athanasiadis	5	Native		R
Rhodophyta	*Gastroclonium ovatum* (Hudson) Papenfuss	2	Native	New record	R
Rhodophyta	*Gastroclonium reflexum* (Chauvin) Kützing	20	Native		C
Rhodophyta	*Gayliella flaccida* (Harvey ex Kützing) T.O.Cho & L.J.McIvor	4	Native		R
Rhodophyta	*Gelidium arbuscula* Bory ex Børgesen	4	Native	New record	R
Rhodophyta	*Gelidium microdon* Kützing	81	Native		A
Rhodophyta	*Gelidium pusillum* (Stackhouse) Le Jolis	76	Native	New record	A
Rhodophyta	*Gelidium spinosum* (S.G.Gmelin) P.C.Silva	35	Native		C
Rhodophyta	*Gigartina pistillata* (S.G.Gmelin) Stackhouse	2	Native	New record	R
Rhodophyta	*Gracilariopsis longissima* (S.G.Gmelin) Steentoft, L.M.Irvine & Farnham	1	Native	New record	R
Rhodophyta	*Grateloupia filicina* (J.V.Lamouroux) C.Agardh	3	Native	New record	R
Rhodophyta	*Gymnogongrus crenulatus* (Turner) J.Agardh	38	Native	New record	C
Rhodophyta	*Gymnogongrus griffithsiae* (Turner) C.Martius	37	Native	New record	C
Rhodophyta	*Gymnothamnion elegans* (Schousboe ex C.Agardh) J.Agardh	2	Native	New record	R
Rhodophyta	*Halarachnion ligulatum* (Woodward) Kützing	2	Native	New record	R
Rhodophyta	*Herposiphonia secunda* (C.Agardh) Ambronn	2	Native		R
Rhodophyta	*Heterosiphonia crispella* (C.Agardh) M.J.Wynne	5	Native	New record	R
Rhodophyta	*Hildenbrandia crouaniorum* J.Agardh	2	Native	New record	R
Rhodophyta	*Hypnea arbuscula* P.J.L.Dangeard	1	Native		R
Rhodophyta	*Hypnea cervicornis* J.Agardh	2	Native		R
Rhodophyta	*Hypnea flagelliformis* Greville ex J.Agardh	8	Introduced		R
Rhodophyta	*Hypnea musciformis* (Wulfen) J.V.Lamouroux	97	Uncertain		A
Rhodophyta	*Hypnea spinella* (C.Agardh) Kützing	25	Native	New record	C
Rhodophyta	*Hypoglossum hypoglossoides* (Stackhouse) Collins & Hervey	2	Native	New record	R
Rhodophyta	*Jania capillacea* Harvey	16	Native	New record	C
Rhodophyta	*Jania longifurca* Zanardini	9	Uncertain		R
Rhodophyta	Jania pedunculata var. adhaerens (J.V.Lamouroux) A.S.Harvey, Woelkerling & Reviers	8	Native	New record	R
Rhodophyta	*Jania pumila* J.V.Lamouroux	10	Native	New record	C
Rhodophyta	*Jania rubens* (Linnaeus) J.V.Lamouroux	43	Native		C
Rhodophyta	*Jania virgata* (Zanardini) Montagne	13	Uncertain		C
Rhodophyta	*Kallymenia reniformis* (Turner) J.Agardh	1	Native	New record	R
Rhodophyta	*Laurencia brongniartii* J.Agardh	3	Introduced		R
Rhodophyta	*Laurencia chondrioides* Børgesen	11	Introduced		C
Rhodophyta	*Laurencia dendroidea* J.Agardh	10	Introduced		C
Rhodophyta	*Laurencia intricata* J.V.Lamouroux	2	Native	New record	R
Rhodophyta	*Laurencia microcladia* Kützing	3	Native	New record	R
Rhodophyta	*Laurencia minuta* Vandermeulen, Garbary & Guiry	18	Introduced	New record	C
Rhodophyta	*Laurencia obtusa* (Hudson) J.V.Lamouroux	2	Native		R
Rhodophyta	*Laurencia pyramidalis* Bory ex Kützing	11	Native	New record	C
Rhodophyta	*Laurencia tenera* C.K.Tseng	17	Native	New record	C
Rhodophyta	*Laurencia viridis* Gil-Rodríguez & Haroun	4	Macaronesian endemism	New record	R
Rhodophyta	*Laurenciella marilzae* (Gil-Rodríguez, Sentíes, Díaz-Larrea, Cassano & M.T.Fujii) Gil-Rodríguez, Sentíes, Díaz-Larrea, Cassano & M.T.Fujii	2	Native	New record	R
Rhodophyta	*Lejolisia mediterranea* Bornet	1	Native	New record	R
Rhodophyta	*Leptosiphonia brodiei* (Dillwyn) A.M.Savoie & G.W.Saunders	2	Uncertain		R
Rhodophyta	*Liagora viscida* (Forsskål) C.Agardh	8	Native	New record	R
Rhodophyta	*Lithophyllum incrustans* Philippi	1	Native		R
Rhodophyta	*Lomentaria articulata* (Hudson) Lyngbye	75	Native	New record	A
Rhodophyta	*Lomentaria clavellosa* (Lightfoot ex Turner) Gaillon	1	Uncertain		R
Rhodophyta	*Lophosiphonia cristata* Falkenberg	13	Native		C
Rhodophyta	*Meredithia microphylla* (J.Agardh) J.Agardh	10	Native	New record	C
Rhodophyta	*Nemalion elminthoides* (Velley) Batters	21	Native		C
Rhodophyta	*Nitophyllum punctatum* (Stackhouse) Greville	11	Native	New record	C
Rhodophyta	*Osmundea hybrida* (A.P.de Candolle) K.W.Nam	5	Native	New record	R
Rhodophyta	*Osmundea oederi* (Gunnerus) G.Furnari	3	Native		R
Rhodophyta	*Osmundea pinnatifida* (Hudson) Stackhouse	23	Native		C
Rhodophyta	*Osmundea truncata* (Kützing) K.W.Nam & Maggs	24	Native	New record	C
Rhodophyta	*Peyssonnelia squamaria* (S.G.Gmelin) Decaisne ex J.Agardh	8	Native		R
Rhodophyta	*Phyllophora gelidioides* P.Crouan & H.Crouan ex Karsakoff	1	Native	New record	R
Rhodophyta	*Phymatolithon calcareum* (Pallas) W.H.Adey & D.L.McKibbin ex Woelkering & L.M.Irvine	1	Native		R
Rhodophyta	*Platoma cyclocolpum* (Montagne) F.Schmitz	9	Native		R
Rhodophyta	*Plocamium cartilagineum* (Linnaeus) P.S.Dixon	28	Native		C
Rhodophyta	*Polysiphonia atlantica* Kapraun & J.N.Norris	3	Native	New record	R
Rhodophyta	*Polysiphonia ceramiiformis* P.Crouan & H.Crouan	1	Native	New record	R
Rhodophyta	*Polysiphonia havanensis* Montagne	3	Native	New record	R
Rhodophyta	*Polysiphonia opaca* (C.Agardh) Moris & De Notaris	3	Native		R
Rhodophyta	*Polysiphonia stricta* (Mertens ex Dillwyn) Greville	6	Native		R
Rhodophyta	*Porphyra umbilicalis* Kützing	1	Native	New record	R
Rhodophyta	*Pterocladiella capillacea* (S.G.Gmelin) Santelices & Hommersand	131	Native		A
Rhodophyta	*Rhodymenia holmesii* Ardissone	22	Native	New record	C
Rhodophyta	*Rhodymenia pseudopalmata* (J.V.Lamouroux) P.C.Silva	9	Native	New record	R
Rhodophyta	*Schizymenia apoda* (J.Agardh) J.Agardh	1	Native		R
Rhodophyta	*Schottera nicaeensis* (J.V.Lamouroux ex Duby) Guiry & Hollenberg	1	Uncertain		R
Rhodophyta	*Scinaia interrupta* (A.P.de Candolle) M.J.Wynne	2	Native		R
Rhodophyta	*Sebdenia dichotoma* Berthold	7	Native		R
Rhodophyta	*Sebdenia rodrigueziana* (Feldmann) Codomier ex Parkinson	6	Native		R
Rhodophyta	*Spermothamnion repens* (Dillwyn) Magnus	1	Native	New record	R
Rhodophyta	*Sphaerococcus coronopifolius* Stackhouse	16	Native	New record	C
Rhodophyta	*Spongoclonium caribaeum* (Børgesen) M.J.Wynne	4	Introduced		R
Rhodophyta	*Spyridia filamentosa* (Wulfen) Harvey	2	Native	New record	R
Rhodophyta	*Symphyocladia marchantioides* (Harvey) Falkenberg	44	Introduced		C
Rhodophyta	*Taenioma nanum* (Kützing) Papenfuss	3	Native		R
Rhodophyta	*Tenarea tortuosa* (Esper) Me.Lemoine	1	Native		R
Rhodophyta	*Vertebrata fruticulosa* (Wulfen) Kuntze	4	Native	New record	R
Rhodophyta	*Vertebrata fucoides* (Hudson) Kuntze	1	Uncertain	New record	R
Rhodophyta	*Vertebrata furcellata* (C.Agardh) Kuntze	1	Native		R
Rhodophyta	*Vertebrata reptabunda* (Suhr) Díaz-Tapia & Maggs	10	Uncertain		C
Rhodophyta	*Vertebrata tripinnata* (Harvey) Kuntze	1	Native		R
Rhodophyta	*Wrangelia penicillata* (C.Agardh) C.Agardh	3	Native	New record	R
Rhodophyta	*Yuzurua poiteaui* (J.V.Lamouroux) Martin-Lescanne	4	Native	New record	R
Chlorophyta	*Anadyomene saldanhae* A.B.Joly & E.C.Oliveira	94	Native	New record	A
Chlorophyta	*Anadyomene stellata* (Wulfen) C.Agardh	16	Uncertain		C
Chlorophyta	*Blidingia minima* (Nägeli ex Kützing) Kylin	4	Native		R
Chlorophyta	*Bryopsis cupressina* J.V.Lamouroux	4	Native	New record	R
Chlorophyta	*Bryopsis pennata* J.V.Lamouroux	1	Native	New record	R
Chlorophyta	*Bryopsis plumosa* (Hudson) C.Agardh	6	Native	New record	R
Chlorophyta	*Chaetomorpha aerea* (Dillwyn) Kützing	8	Native	New record	R
Chlorophyta	*Chaetomorpha ligustica* (Kützing) Kützing	1	Native	New record	R
Chlorophyta	*Chaetomorpha linum* (O.F.Müller) Kützing	4	Native		R
Chlorophyta	*Chaetomorpha pachynema* (Montagne) Kützing	84	Native		A
Chlorophyta	*Cladophora albida* (Nees) Kutzing	6	Native	New record	R
Chlorophyta	*Cladophora coelothrix* Kützing	32	Native	New record	C
Chlorophyta	*Cladophora conferta* P.Crouan & H.Crouan	4	Native		R
Chlorophyta	*Cladophora hutchinsiae* (Dillwyn) Kützing	2	Native	New record	R
Chlorophyta	*Cladophora laetevirens* (Dillwyn) Kützing	7	Uncertain		R
Chlorophyta	*Cladophora lehmanniana* (Lindenberg) Kützing	8	Native	New record	R
Chlorophyta	*Cladophora liebetruthii* Grunow	2	Native	New record	R
Chlorophyta	*Cladophora prolifera* (Roth) Kützing	43	Native		C
Chlorophyta	*Cladophoropsis macromeres* W.R.Taylor	2	Native	New record	R
Chlorophyta	*Cladophoropsis membranacea* (Hofman Bang ex C.Agardh) Børgesen	6	Uncertain		R
Chlorophyta	*Codium adhaerens* C.Agardh	101	Native		A
Chlorophyta	*Codium decorticatum* (Woodward) M.Howe	16	Native		C
Chlorophyta	*Codium effusum* (Rafinesque) Delle Chiaje	22	Uncertain		C
Chlorophyta	*Codium elisabethiae* O.C.Schmidt	39	Macaronesian endemism		C
Chlorophyta	Codium fragile subsp. fragile (Suringar) Hariot	4	Introduced	New record	R
Chlorophyta	*Codium taylorii* P.C.Silva	3	Native		R
Chlorophyta	*Codium vermilara* (Olivi) Delle Chiaje	18	Native	New record	C
Chlorophyta	*Ernodesmis verticillata* (Kützing) Børgesen	2	Native	New record	R
Chlorophyta	*Lychaete pellucida* (Hudson) M.J.Wynne	10	Native	New record	C
Chlorophyta	*Microdictyon umbilicatum* (Velley) Zanardini	11	Native	New record	C
Chlorophyta	*Pseudorhizoclonium africanum* (Kützing) Boedeker	1	Native	New record	R
Chlorophyta	*Ulva clathrata* (Roth) C.Agardh	14	Native		C
Chlorophyta	*Ulva compressa* Linnaeus	13	Native		C
Chlorophyta	*Ulva intestinalis* Linnaeus	13	Native		C
Chlorophyta	*Ulva lactuca* Linnaeus	1	Uncertain		R
Chlorophyta	*Ulva linza* Linnaeus	3	Native		R
Chlorophyta	*Ulva polyclada* Kraft	5	Native		R
Chlorophyta	*Ulva prolifera* O.F.Müller	3	Native		R
Chlorophyta	*Ulva rigida* C.Agardh	133	Native		A
Chlorophyta	*Valonia macrophysa* Kützing	1	Native	New record	R
Chlorophyta	*Valonia utricularis* (Roth) C.Agardh	7	Native	New record	R
Ochrophyta	*Ascophyllum nodosum* (Linnaeus) Le Jolis	4	Native		R
Ochrophyta	*Bachelotia antillarum* (Grunow) Gerloff	4	Native		R
Ochrophyta	*Canistrocarpus cervicornis* (Kützing) De Paula & De Clerck	1	Native	New record	R
Ochrophyta	*Carpomitra costata* (Stackhouse) Batters	4	Native	New record	R
Ochrophyta	*Cladostephus spongiosum* (Hudson) C.Agardh	6	Native	New record	R
Ochrophyta	*Colpomenia sinuosa* (Mertens ex Roth) Derbès & Solier	98	Native		A
Ochrophyta	*Cutleria multifida* (Turner) Greville	9	Uncertain		R
Ochrophyta	*Cutleria multifida* (Turner) Greville, phase *Aglaozonia parvula* (Greville) Zanardini	10	Uncertain	New record	C
Ochrophyta	*Cystoseira compressa* (Esper) Gerloff & Nizamuddin	23	Native	New record	C
Ochrophyta	*Cystoseira foeniculacea* (Linnaeus) Greville	2	Native	New record	R
Ochrophyta	*Cystoseira humilis* Schousboe ex Kützing	5	Native		R
Ochrophyta	*Dictyopteris polypodioides* (A.P.de Candolle) J.V.Lamouroux	1	Native	New record	R
Ochrophyta	*Dictyota bartayresiana* J.V.Lamouroux	3	Native	New record	R
Ochrophyta	*Dictyota cyanoloma* Tronholm, De Clerck, A.Gómez-Garreta & Rull Lluch	3	Native		R
Ochrophyta	*Dictyota dichotoma* (Hudson) J.V.Lamouroux	35	Native		C
Ochrophyta	Dictyota dichotoma var. intricata (C.Agardh) Greville	7	Native	New record	R
Ochrophyta	*Dictyota implexa* (Desfontaines) J.V.Lamouroux	6	Native	New record	R
Ochrophyta	*Ectocarpus fasciculatus* Harvey	2	Native		R
Ochrophyta	*Fucus spiralis* Linnaeus	32	Uncertain		C
Ochrophyta	*Halopteris filicina* (Grateloup) Kützing	77	Native		A
Ochrophyta	*Halopteris scoparia* (Linnaeus) Sauvageau	129	Native		A
Ochrophyta	*Hincksia ovata* (Kjellman) P.C.Silva	1	Native		R
Ochrophyta	*Hydroclathrus clathratus* (C.Agardh) M.Howe	2	Native	New record	R
Ochrophyta	*Hydroclathrus tilesii* (Endlicher) Santiañez & Wynne	15	Introduced	New record	C
Ochrophyta	*Leathesia marina* (Lyngbye) Decaisne	2	Uncertain	New record	R
Ochrophyta	*Lobophora variegata* (J.V.Lamouroux) Womersley ex E.C.Oliveira	2	Native		R
Ochrophyta	*Microzonia floridana* (E.C.Henry) Camacho & Fredericq	3	Native		R
Ochrophyta	*Myrionema strangulans* Greville	2	Native	New record	R
Ochrophyta	*Nemoderma tingitanum* Schousboe ex Bornet	39	Native	New record	C
Ochrophyta	*Padina pavonica* (Linnaeus) Thivy	91	Native		A
Ochrophyta	*Petalonia binghamiae* (J.Agardh) K.L.Vinogradova	10	Introduced		C
Ochrophyta	*Petrospongium berkeleyi* (Greville) Nägeli ex Kützing	1	Native	New record	R
Ochrophyta	*Sargassum cymosum* C.Agardh	5	Native		R
Ochrophyta	*Sargassum desfontainesii* (Turner) C.Agardh	2	Native	New record	R
Ochrophyta	*Sargassum furcatum* Kützing	25	Native	New record	C
Ochrophyta	*Sargassum vulgare* C.Agardh	1	Native		R
Ochrophyta	*Scytosiphon lomentaria* (Lyngbye) Link	1	Native	New record	R
Ochrophyta	*Sphacelaria cirrosa* (Roth) C.Agardh	2	Native		R
Ochrophyta	*Sphacelaria tribuloides* Meneghini	1	Uncertain		R
Ochrophyta	*Spongonema tomentosum* (Hudson) Kützing	1	Native	New record	R
Ochrophyta	*Taonia atomaria* (Woodward) J.Agardh	2	Native	New record	R
Ochrophyta	*Treptacantha abies-marina* (S.G.Gmelin) Kützing	72	Native	New record	A
Ochrophyta	*Zonaria tournefortii* (J.V.Lamouroux) Montagne	90	Native		A

**Table 3.** Main taxonomic figures with information on the species origin and status.

Phyllum	Order	Family	Specimens Number	Total taxa	Total species	Native	Introduced	Uncertain	Macaronesian endemism	New record
Rhodophyta	15	39	2404	197	142	113	11	16	2	69
Chlorophyta	3	10	773	53	41	34	1	5	1	20
Ochrophyta	12	18	866	53	42	36	2	4		21
Total	30	67	4043	303	225	183	14	25	3	110
